# Serendipitous Isolation of Non-*Vibrio* Bacterial Strains Carrying the Cholera Toxin Gene from Environmental Waters in Indonesia

**DOI:** 10.1155/2013/406078

**Published:** 2013-12-03

**Authors:** Yusuke Shibata, Ryohei Nomoto, Garry Cores de Vries, Ro Osawa

**Affiliations:** ^1^Department of Bioresource Science, Graduate School of Agricultural Science, Kobe University, Rokko-dai 1-1, Nada-ku, Kobe 657-8501, Japan; ^2^Department of Veterinary Public Health, Faculty of Veterinary Medicine, Airlangga University, Raya Mulyorejo, Surabaya 60115, Indonesia; ^3^Research Center for Food Safety and Security, Graduate School of Agricultural Science, Kobe University, Rokko-dai 1-1, Nada-ku, Kobe 657-8501, Japan

## Abstract

We initially attempted to isolate a *Vibrio cholerae* O1 El Tor biotype that carries a novel variant of the cholera toxin gene (*ctxAB*) from environmental waters of Indonesia, where the seventh cholera pandemic by *V. cholerae* O1 El Tor biotype began. Nested PCR targeting the gene revealed that a total of eight strains were found to carry *ctxAB*. However, sequencing of the 16S rRNA genes of these isolates showed they were not *V. cholerae* but were either *Klebsiella*, *Enterobacter*, *Pantoea*, or *Aeromonas*. Subsequent nested PCR assays targeting all genes known to be encoded on the CTX phage (i.e., *zot*, *ace*, *orfU*, *cep*, *rstB*, *rstA*, and *rstR*) showed that one isolate belonged to the *Enterobacter* genus carried all the genes tested, while the other isolates lacked either 2, 3, or 5 of the genes. This evidence suggests that phages with *ctxAB* are genetically diverse and can infect not only *V. cholerae* and *V. mimicus* but also other species and genera in the form of a pseudolysogen.

## 1. Introduction

*Vibrio cholerae* is a gastrointestinal pathogen that causes cholera, a notorious enteric disease with serious morbidity and mortality worldwide. The clinical strains belonging to serogroups O1 and O139 are responsible for all the major cholera epidemics and pandemics on record. The main virulence factor causing the disease, cholera toxin (CTX), is encoded by *ctxA* and *ctxB* (*ctxA *and *ctxB*; collectively referred to as *ctxAB*) [1]. The *ctxAB* genes are present on a filamentous phage, called CTX phage, which has been shown to lysogenize only *V. cholerae*, *V. mimicus*, and other *Vibrio* species [2]. To date, the world has experienced seven major pandemics of cholera since the early 19th century. The 5th and 6th pandemics were caused by toxigenic strains belonging to the classical biotype of serogroup O1 that possesses the classical type of *ctxB,* whereas the ongoing 7th pandemic, which began in 1961 on the island of Sulawesi in Indonesia [1], is caused by the El Tor biotype that carries the El Tor type of *ctxB* that has an amino acid sequence slightly different from that of the classical type [3].

Over the past two decades, several *V. cholerae* strains of different serogroups carrying *ctxB *with amino acid sequences slightly different from each other have emerged. As of 2009, a total of nine genomic variants of* ctxB* including the classical type have been reported [4]. We therefore attempted to isolate the *V. cholerae* O1 El Tor biotype that carried the novel *ctxB* variant gene from environmental waters of Indonesia. During the course of this attempt, we isolated bacterial strains that also carried *ctxB* but did not taxonomically belong to the genus *Vibrio*. This paper reports the phenotypic and genotypic characteristics of these isolates.

## 2. Materials and Methods

### 2.1. Isolation of *V. cholerae* O1 El Tor Like Strains

Water samples were collected from various sources such as rivers, estuaries, and seas in Indonesia (i.e., coastal areas from Surabaya to Bali Island). Approximately 20 mL of each sample was mixed with equal volumes of double strength alkaline peptone water (APW, Oxoid Ltd., Basingstoke, UK) and incubated for 48 h at room temperature (ca. 24°C). After enrichment, a 10 *μ*L aliquot of culture was streaked on a polymyxin B mannose tellurite (PMT) agar plate (Nissui Pharmaceutical Co., Ltd., Tokyo, Japan) for the preferential isolation of *V. cholerae *O1 El Tor, followed by incubation at room temperature (at ca. 24°C) for 48 h. After incubation, well-isolated yellowish colonies resembling those of the reference *V. cholerae *O1 El Tor strains 5H332 and 18H24 (kind gift of Osaka Prefectural Institute of Public Health, Japan) on the PMT plates were selected as “the El Tor variant” candidate isolates.

### 2.2. Screening for ctxA and ctxB Carrying Isolates

The candidate isolates were then screened for possession of *ctxA *and* ctxB* by conventional PCR assay. The sets of primer sequences are listed in Table 1. Davis and Waldor [5] showed that CTX phage DNA can be present as either single-stranded DNA, double-stranded plasmid-like DNA extra chromosomally, or double-stranded linear DNA as part of the chromosome in the lysogenic state. On this basis, genomic and plasmid DNA of the isolates were extracted using a commercially available whole genome DNA extraction kit (illustra genomicPrep Mini Spin Kit, GE Healthcare) and plasmid DNA extraction kit (illustra plasmidPrep Mini Spin Kit, GE Healthcare, Little Chalfont, UK), respectively. To increase the sensitivity for gene detection, we performed nested PCR assays using the diluted 1st-PCR products as template DNA. Nested PCR products obtained were purified using a High Pure PCR Product purification kit (Roche Diagnostics GmbH, Mannheim, Germany) and sequenced using a BigDye Terminator v3.1 cycle sequencing kit (Applied Biosystems, Warrington, UK), according to the manufacturer's instructions. Sequencing products were read on an ABI Prism 3100 genetic analyzer (Applied Biosystems, Darmstadt, Germany).

### 2.3. Genetic and Biochemical Identification

The isolates carrying the intact *ctxA* and *ctxB* were then subjected to taxonomic identification, based on sequencing of their 16S rRNA genes using a commercial kit (Bacterial 16S rDNA PCR Kit, Takara Bio Inc., Kyoto, Japan) and their biochemical characteristics using a commercial identification kit (API 20E, Biomerieux Inc., Marcy-l'Etoile, France).

### 2.4. Detection of Other CTX Phage Encoded Genes and the Genes Encoding the Phage Receptors

In addition to *ctxAB*, the CTX phage genome also includes genes required for phage morphogenesis, replication, regulation of phage gene expression, and phage integration (i.e.,* zot*, *ace*, *orfU*, *cep*, *rstB*, *rstA*, and *rstR*) [6]. We, therefore, performed sets of PCR and nested PCR assays targeting these genes using the plasmid DNAs of the eight isolates.

Davis and Waldor [5] showed that the CTX phage can infect bacterial cells through a toxin-coregulated pilus (TCP) and a TolQRA complex, which act as the phage receptor and facilitator of the phage's traverse of the periplasmic space of the host cell, respectively. We therefore performed PCR assays using genomic DNA of the isolates targeting the genes associated with TCP (i.e.,* tcpA*) and the complex (i.e., *tolQ*, *tolR*, and *tolA*, collectively referred to as *tolQRA*) of *V. cholerae* N16961 [7].

### 2.5. S1 Nuclease Treatment

It was described that CTX phage DNA can be present as either single-stranded DNA, double-stranded plasmid-like DNA extra chromosomally, or double-stranded linear DNA as part of the chromosome in the lysogenic state [5]. On this basis, we treated the plasmid DNA of GCDV 10-1 and the genomic DNA of *V. cholerae* N16961 (as a control) with or without S1 nuclease (Takara Bio) to specifically degrade single-stranded nucleic acids. We then performed a PCR targeting *ctxA*.

### 2.6. CT Production

To examine the production of CT, we performed a reverse, passive latex agglutination (RPLA) test (VET-RPLA; Denka Seiken Co., Ltd. in Tokyo, Japan) on the *ctxAB*-positive isolates according to the method recommended by the manufacturers.

## 3. Results and Discussion

**Figure 1:**
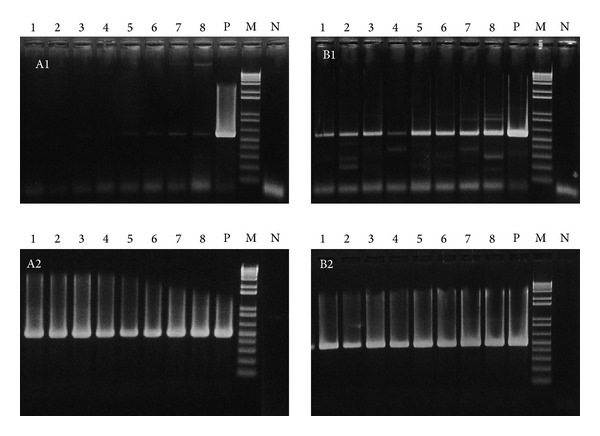
Comparison of PCR and nested PCR targeting *ctxA *with template DNA prepared from a commercial whole genome DNA extraction kit ((A1), 1st-PCR; (A2), nested PCR) and a commercial plasmid DNA extraction kit ((B1), 1st-PCR; (B2), nested PCR). Lane 1, GCDV 10-1; lane 2, GCDV 10-2; lane 3, GCDV 31-1; lane 4, GCDV 34-3; lane 5, GCDV 47-1; lane 6, GCDV 28-2; lane 7, GCDV 37-1; lane 8, GCDV 50-6; lane P, *Vibrio cholerae* N16961 (positive control); lane M, 100 bp ladder size marker; lane N, PCR negative control.

Through our isolation attempt, a total of 54 candidate isolates that showed *V. cholerae* O1 El Tor like colony appearance were obtained. The candidate isolates were then screened for possession of *ctxA* and *ctxB* by conventional PCR assay. The PCR of genomic DNA from the isolates yielded barely visible amplicons on the gel, whereas the plasmid DNA preparation of the eight isolates showed clearly visible amplicons of the expected size (Figure 1). Nested PCR of both the genomic and plasmid DNA preparations revealed clearly visible amplicons of the expected size (Figure 1). We then sequenced the nested PCR products to determine both the nucleotide and deduced amino acid sequences and found that they were matched exactly with the sequences of the El Tor biotype CT [8] (100% similarity, data not shown).

We then performed a PCR-based method for identification of *V. cholerae *[9] or *V. mimicus *[10]. However, all eight isolates were negative for both PCRs (data not shown). We therefore determined the taxonomic identities of the isolates by sequencing of their 16S rRNA genes and their biochemical characteristics (API 20E). The sequencing results showed that one isolate, GCDV 50-6, had a similarity level >99% with *V. parahaemolyticus* (Table 2). The other isolates were assigned to either the genus *Klebsiella*, *Enterobacter*, *Pantoea*, or *Aeromonas *(Table 2). Taxa identified by the API 20E showed that none of the isolates belonged to *V. cholerae*, with six being either *Klebsiella*, *Enterobacter*, *Aeromonas*, or *V. fluvialis* species with a high degree (>97%) of certainty (Table 2). These findings suggest that the CTX phage can infect not only *V. cholerae* and *V. mimicus* but also other species and genera in the form of pseudolysogen. In pseudolysogeny, phage DNA replication is not synchronized with the cell cycle, as opposed to the synchronization that occurs during lysogeny. This leads to unstable coexistence of the phage in the cell, which results in a high proportion of cured cells over several generations [11]. This situation has been well described in *V. harveyi*, in which the proportion of phage DNAs relative to host DNA rapidly decreased after serial subcultures in Mueller-Hinton broth [12]. We therefore subcultured the isolates in APW at 37°C at approximately three-day intervals over four serial cultures. PCR and nested PCR assays targeting* ctxA* were performed using plasmid DNA prepared from the initial and final subculture. We found that successive subculturing resulted in the disappearance of the targeted PCR amplicons (data not shown).

**Figure 2:**
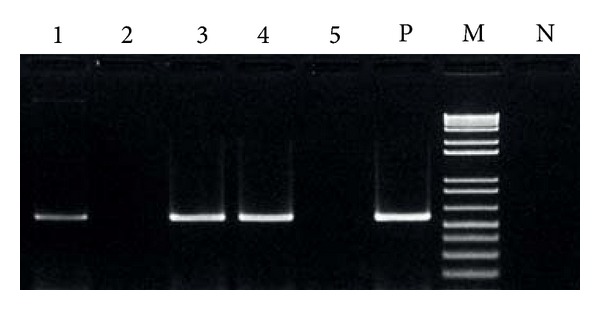
The result of PCR targeting *ctxA *using the plasmid DNA from GCDV 10-1 and the genomic DNA from *V. cholerae* N16961 (as control), with or without S1 nuclease treatment. Lane 1, GCDV 10-1 without S1 nuclease treatment; lane 2, GCDV 10-1 with S1 nuclease treatment; lane 3, *V. cholerae* N16961 without S1 nuclease treatment; lane 4, *V. cholerae* N16961 with S1 nuclease; lane 5, *V. cholerae* N16961 single-stranded DNA prepared by rapid cooling after heat denaturation with S1 nuclease; lane 6, *V. cholerae* N16961 (positive control); lane M, 100 bp ladder size marker; lane P, PCR-negative control.

If the CTX phage-like genetic elements are present in the form of pseudolysogeny, they may be single-stranded DNA or double-stranded plasmid-like DNA extra chromosomally [5]. The PCR of the plasmid DNA of GCDV 10-1 treated with S1 nuclease failed to yield any amplicons (Figure 2). This indicated that the phage is not lysogenized in the host chromosome but is present as single-stranded DNA in very few host cells. However, slight DNA bands of amplicons appeared on the gel of genomic DNA extracts samples (Figure 1 (A1)). It is suggested that there is the contamination of plasmid DNA in genomic DNA extracts during the course of genomic DNA extraction.

If CTX phage infects these eight isolates, they also carry CTX phage encoded genes other than *ctxAB* (i.e., *zot*, *ace*, *orfU*, *cep*, *rstB*, *rstA*, and *rstR*) and genes encoding CTX phage receptors (i.e., *tolQ*, *tolR*, *tolA* and *tcpA*). The results of nested PCR targeting CTX phage encoded genes showed that only one isolate, GCDV 28-2, was positive for all the genes tested, while the other isolates lacked either 2, 3, or 5 of the genes (Table 2). This suggests that the genetic elements of the CTX phage are not entirely homogenic to those of the known CTX phage but have appreciable variation in genetic content. The results of PCR targeting the genes encoding the phage receptors showed that all isolates were negative for *tcpA*, *tolQ*, *tolR*, and *tolA*. The findings suggest that the isolates acquired the CTX-like phage through a TCP-independent, or possibly a TolQRA-independent mechanism, similar to that demonstrated *in vitro* by Boyd and Waldor [13]. However, we cannot verify the absence of *tcpA* and *tolQRA* genes by PCR alone. As of 2011, Kumar et al. [14] reported that there were several variants of the *tcpA* gene which could be classified into 10 genetically distinct clusters in *V. cholerae* and *V. mimicus.* Therefore, it remains to be determined whether these isolates carry *tcpA* and *tolQRA *by not PCR but southern blotting analysis.

The results of examination of CT production showed that all of the eight isolates were negative, indicating that none of them produced CT. The expression of the virulence genes are regulated by the transcriptional regulator ToxR, which binds to a repeated heptanucleotide motif (TTTTGAT), localized between the *zot* and *ctxA* genes, to activate the transcription of the *ctxAB* genes [15, 16]. Epidemic strains of serogroups O1 and O139 possess three or more copies of this heptanucleotide, but those which did not produce CT possessed only two copies [17]. On this basis, these 8 *ctxAB*-positive isolates may not have met such requirements to produce detectable CT.

It has long been believed that the CTX phage almost exclusively infects *V. cholerae* and *V. mimicus *[18]. However, Sechi et al. [19] reported that the *V. cholerae* virulence genes were widely distributed among different *Vibrio* species. More recently, Snoussi et al. [20] reported a scattered distribution of CTX phage-encoded genes including *ctxA* in many strains of *V. alginolyticus *isolated from seawater and molluscs. The present study is therefore the first to report that bacterial strains carrying *ctxAB* are present not only in the genus *Vibrio *but also in other genera in aquatic environments. The study also demonstrated that nested PCR is a useful method for advancing research on pseudolysogeny in environmental samples. The mechanism by which the non-*Vibrio* strains acquire *ctxAB* remains to be determined.

## Figures and Tables

**Table 1:** Primer sequences used for the sets of PCR amplification. All primers were designed on the basis of the sequence of *V. cholerae *O1 El Tor N16961 (Genbank accession no. AE003852).

Genes	Usage	Sequence	
		Forward (5′-3′)	Reverse (5′-3′)
*ctxB *	1st-PCR nested PCR	AGCATATGCACATGGAACACC TGTGTGCAGAATACCACAACA	AATTGCGGCAATCGCATGA GCAATCCTCAGGGTATCCTT
*ctxA *	1st-PCR nested PCR	CGGGCAGATTCTAGACCTCCTG GCAGTCAGGTGGTCTTATGCC	CGATGATCTTGGAGCATTCCCAC GCATGATGAATCCACGGCTC
*zot *	1st-PCR nested PCR	TCGCTTAACGATGGCGCGTTTT GATGAATGTGGTCGCATCTGG	AACCCCGTTTCACTTCTACCCA ATCCGGTAACGGTAGCACCTT
*ace *	1st-PCR nested PCR	TAAGGATGTGCTTATGATGGACACCC	CGTGATGAATAAAGATACTCATAGG
		TGGCTTGTGATCAAGCTCG	CATCAAAGCCTGAAGCACG
*orfU *	1st-PCR nested PCR	GGTGTTATTTGATGGCTGCATG ACTTGTCCGGAGCATCTGCA	AAACATCAATGCGGATTTCCTC TGACTGCGGTGACACTCTCAA
*cep *	1st-PCR nested PCR	GCTACATGTTTAGCTCACTG	TTTAGCCTTACGAATTAAGCC
		GCTTTCACTCGGGGTTTTCT	TCAGAACAATTGCCCCCA
*rstB *	1st-PCR nested PCR	CTCTCATTCTGAAGGGGTGAGT GTTTGACGTTTGGCTTGCAG	AGGCTTATCCAATGGCTTGC AGGTTGCGTGATGGGTCTT
*rstA *	1st-PCR nested PCR	GCAGATTTTCACTCTTGACGAA GAAGTGGAACGTTGCCATGA	GGTTGAGTGAATCGTCGTGAAT CACCTTGACAGGCAAGGAAT
*rstR *	1st-PCR nested PCR	CTAGCCAACCAAAGAAAGGCA GGGGAAGGTTTGCCTACAAT	GCACCATGATTTAAGATGCTCTTG CCCATCTTCCGCATAGTTCA
*tcpA *	PCR	AGAAAACCGGTCAAGAGGG	CTGTGAATGGAGCAGTTCCTG
*tolQ *	PCR	TCGTGGGCGGCAATTATCA	AGCCATCACTTGACGGTGGAG
*tolR *	PCR	TGGCTGGCTATCAAACCAA	TTTAAGGTCCGTGAGTAGCCC
*tolA *	PCR	CGGTGCTTTGGTCGCGATAT	CGTCAGGTTGATCTTTCGGCA

**Table 2:** Tentative taxonomic identity of cholera toxin gene carrying environmental bacterial isolates based on their 16S rRNA gene sequences and biochemical characteristics and distribution of CTX phage encoded genes in each isolate.

Strain No.	Taxonomic Identity by 16S rRNA gene sequence (% identity)*	Taxon phenotypically identified by a commercial kit (API 20E) (% probability of identity)	Results of nested PCR targeting genes encoded in the CTX phage								
			*rstR *	*rstA *	*rstB *	*cep *	*orfU *	*ace *	*zot *	*ctxA *	*ctxB *
GCDV 10-1	*Klebsiella variicola* (99%, 1317 bp/1318 bp)	*Klebsiella pneumoniae* (99.92%)	+	−	−	+	−	+	+	+	+
GCDV 10-2	*Pantoea agglomerans* (99%, 1312 bp/1317 bp)	*Cirtobacter diversus* (60.30%)	−	−	−	−	+	−	+	+	+
GCDV 31-1	*Aeromonas cavia*e (99%, 1332 bp/1332 bp)	*Vibrio fluvialis* (99.33%)	−	+	+	+	−	+	+	+	+
GCDV 34-3	*Aeromonas veronii* (100%, 1336 bp/1336 bp)	*Vibrio alginolyticus* (70.67%)	−	−	+	+	+	−	+	+	+
GCDV 47-1	*Enterobacter cloacae* (100%, 1322 bp/1322 bp)	*Enterobacter cloacae* (97.69%)	−	+	+	−	−	−	−	+	+
GCDV 28-2	*Enterobacter hormaechei* (100%, 1304 bp/1304 bp)	*Enterobacter cloacae* (99.42%)	+	+	+	+	+	+	+	+	+
GCDV 37-1	*Aeromonas hydrophila* (100%, 1307 bp/1307 bp)	*Aeromonas hydrophila group* (99.95%)	+	−	+	+	+	−	+	+	+
GCDV 50-6	*Vibrio parahaemolyticus* (99%, 1321 bp/1323 bp)	*Aeromonas salmonicida* (50.43%)	−	−	+	+	+	−	+	+	+
*Vibrio cholerae *N16961 (positive control)	Not determined	Not determined	+	+	+	+	+	+	+	+	+

*Taxa with >98% sequence identities its nearest type strain as determined by comparison of the partial 16S rRNA gene sequence (approx. 1300 bp) of CRIB with sequences present in the database using the BLAST tool from NCBI.

## References

[1] Faruque SM, Albert MJ, Mekalanos JJ (1998). Epidemiology, genetics, and ecology of toxigenic *Vibrio cholerae*. *Microbiology and Molecular Biology Reviews*.

[2] Faruque SM, Mekalanos JJ (2012). Phage-bacterial interactions in the evolution of toxigenic *Vibrio cholerae*. *Virulence*.

[3] Das M, Jaiswal A, Pal S (2012). Dynamics of classical-El Tor switch of *Vibrio cholerae* strains isolated from 1961–2010. *International Journal of Antimicrobial Agents*.

[4] Safa A, Nair GB, Kong RYC (2010). Evolution of new variants of *Vibrio cholerae* O1. *Trends in Microbiology*.

[5] Davis BM, Waldor MK (2003). Filamentous phages linked to virulence of *Vibrio cholerae*. *Current Opinion in Microbiology*.

[6] Boyd EF, Heilpern AJ, Waldor MK (2000). Molecular analyses of a putative CTX*φ* precursor and evidence for independent acquisition of distinct CTX*φ*s by toxigenic *Vibrio cholerae*. *Journal of Bacteriology*.

[7] Heilpern AJ, Waldor MK (2000). CTX*φ* infection of *Vibrio cholerae* requires the *tolQRA* gene products. *Journal of Bacteriology*.

[8] Heidelberg JF, Elsen JA, Nelson WC (2000). DNA sequence of both chromosomes of the cholera pathogen *Vibrio cholerae*. *Nature*.

[9] Chun J, Huq A, Colwell RR (1999). Analysis of 16S-23S rRNA intergenic spacer regions of *Vibrio cholerae* and *Vibrio mimicus*. *Applied and Environmental Microbiology*.

[10] Tarr CL, Patel JS, Puhr ND, Sowers EG, Bopp CA, Strockbine NA (2007). Identification of *Vibrio* isolates by a multiplex PCR assay and *rpoB* sequence determination. *Journal of Clinical Microbiology*.

[11] Ripp S, Miller RV (1998). Dynamics of the pseudolysogenic response in slowly growing cells of *Pseudomonas aeruginosa*. *Microbiology*.

[12] Khemayan K, Pasharawipas T, Puiprom O, Sriurairatana S, Suthienkul O, Flegel TW (2006). Unstable lysogeny and pseudolysogeny in *Vibrio harveyi* siphovirus-like phage 1. *Applied and Environmental Microbiology*.

[13] Boyd EF, Waldor MK (1999). Alternative mechanism of cholera toxin acquisition by *Vibrio cholerae*: generalized transduction of CTXΦ by bacteriophage CP-T1. *Infection and Immunity*.

[14] Kumar P, Thulaseedharan A, Chowdhury G, Ramamurthy T, Thomas S (2011). Characterization of novel alleles of toxin co-regulated pilus a gene (*tcpA*) from environmental isolates of *Vibrio cholerae*. *Current Microbiology*.

[15] Miller VL, Taylor RK, Mekalanos JJ (1987). Cholera toxin transcriptional activator ToxR is a transmembrane DNA binding protein. *Cell*.

[16] Pfau JD, Taylor RK (1996). Genetic footprint of the ToxR-binding site in the promoter for cholera toxin. *Molecular Microbiology*.

[17] Sarkar A, Nandy RK, Nair GB, Ghose AC (2002). Vibrio pathogenicity island and cholera toxin genetic element-associated virulence genes and their expression in non-O1 non-O139 strains of *Vibrio cholerae*. *Infection and Immunity*.

[18] Boyd EF, Moyer KE, Shi L, Waldor MK (2000). Infectious CTXΦ and the *Vibrio* pathogenicity island prophage in *Vibrio mimicus*: evidence for recent horizontal transfer between *V. mimicus* and *V. cholerae*. *Infection and Immunity*.

[19] Sechi LA, Duprè I, Deriu A, Fadda G, Zanetti S (2000). Distribution of *Vibrio cholerae* virulence genes among different *Vibrio* species isolated in Sardinia, Italy. *Journal of Applied Microbiology*.

[20] Snoussi M, Noumi E, Usai D, Sechi LA, Zanetti S, Bakhrouf A (2008). Distribution of some virulence related-properties of *Vibrio alginolyticus* strains isolated from Mediterranean seawater (Bay of Khenis, Tunisia): investigation of eight *Vibrio cholerae* virulence genes. *World Journal of Microbiology and Biotechnology*.

